# Robot-Assisted Gait Self-Training: Assessing the Level Achieved

**DOI:** 10.3390/s21186213

**Published:** 2021-09-16

**Authors:** Andrea Scheidig, Benjamin Schütz, Thanh Quang Trinh, Alexander Vorndran, Anke Mayfarth, Christian Sternitzke, Eric Röhner, Horst-Michael Gross

**Affiliations:** 1 Neuroinformatics and Cognitive Robotics Lab, Ilmenau University of Technology, 98684 Ilmenau, Germany; benjamin.schuetz@tu-ilmenau.de (B.S.); thanh.q.trinh@tediro.com (T.Q.T.); Alexander.Vorndran@tu-ilmenau.de (A.V.); 2 MetraLabs GmbH, 98693 Ilmenau, Germany; anke.mayfarth@tediro.de; 3 tediro GmbH, 98693 Ilmenau, Germany; christian.sternitzke@tediro.com; 4 Orthopedic Department of the Waldkliniken Eisenberg, University Hospital Jena, 07607 Eisenberg, Germany; e.roehner@waldkliniken-eisenberg.de

**Keywords:** robot-assisted gait training, self-training, feedback to the patient, autonomous use, real clinical environment conditions

## Abstract

This paper presents the technological status of robot-assisted gait self-training under real clinical environment conditions. A successful rehabilitation after surgery in hip endoprosthetics comprises self-training of the lessons taught by physiotherapists. While doing this, immediate feedback to the patient about deviations from the expected physiological gait pattern during training is important. Hence, the Socially Assistive Robot (SAR) developed for this type of training employs task-specific, user-centered navigation and autonomous, real-time gait feature classification techniques to enrich the self-training through companionship and timely corrective feedback. The evaluation of the system took place during user tests in a hospital from the point of view of technical benchmarking, considering the therapists’ and patients’ point of view with regard to training motivation and from the point of view of initial findings on medical efficacy as a prerequisite from an economic perspective. In this paper, the following research questions were primarily considered: Does the level of technology achieved enable autonomous use in everyday clinical practice? Has the gait pattern of patients who used additional robot-assisted gait self-training for several days been changed or improved compared to patients without this training? How does the use of a SAR-based self-training robot affect the motivation of the patients?

## 1. Introduction

The independent and self-reliable training of patients independent of the therapist in the rehabilitation process is becoming increasingly important in times of scarce financial and human resources in public healthcare systems. Patients usually receive instructions and recommendations from therapeutic staff on how to carry out self-training for time windows when no therapies with therapists take place. Whether patients actually train and perform the exercises accurately during this time remains an open question. In contrast, self-training assisted by a training robot enables patients not only to exercise independently of the presence of a physiotherapist, but also to receive recommendations for correction from the robot, including positive feedback. In this way, training errors are avoided and the progress of the therapy is strengthened. This “controlled” self-training could result in faster rehabilitation and reintegration, which at the same time contribute to relieving the therapeutic staff.

**Figure 1:**
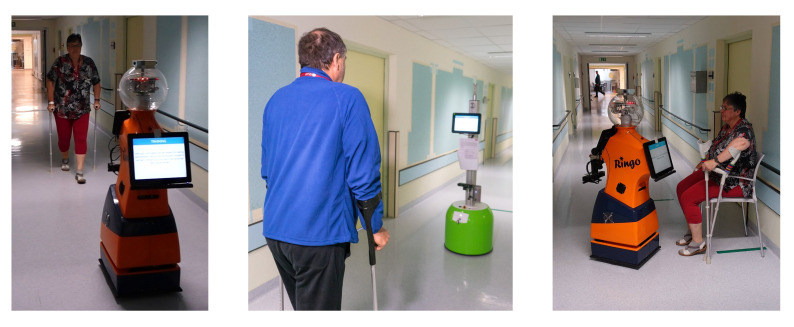
Patients during SAR-based self-training, where training robots drive in front of the patients and provide motivational and corrective feedback on their gait behavior (**left**, **middle**). Patients can pause the training at resting places (**right**).

In the project “Robot-assisted gait training in orthopedic rehabilitation” (ROGER, 2016–2019), a mobile Socially Assistive Robot (SAR)-based self-training robot was developed based on our preliminary work [1], which assists patients after orthopedic operations such as hip or knee replacement surgery in clinical aftercare with personalized gait exercises to restore a gait pattern that is as physiological as possible (see Figure 1). The training robot can observe the patients during gait training by means of its onboard sensor system, is mobile to actively guide them, recognizes errors in the gait training process, intervenes to correct them, and records the course and progress of the training.

The aim of this paper is to present and discuss the results achieved in ROGER during user tests in a hospital from the technical benchmarking point of view, from the therapists’ and patients’ point of view with regard to training motivation, and from the point of view of initial findings on medical efficacy as a prerequisite from an economic perspective.

Special focus was on the following three research questions: Does the level of development achieved enable autonomous use in everyday clinical practice (Question 1)? Did the gait pattern of patients who used additional robot-assisted gait training over several days change or improve compared to patients without this training (Question 2)? How did the use of a training robot affect the patients’ motivation to train (Question 3)?

This paper is based on [2,3,4], which contain concrete details to the results obtained from SAR-assisted gait training and supplements these with considerations of the above issues.

## 2. Requirements

In order to assess the state of development of a technology such as the SAR-based gait self-training discussed in this article, various aspects must be taken into account. These are mainly the technological level of the hardware and software implementation, the usability from the user’s point of view, and the integration into existing structures. Such an assessment can also be made via the Technology Readiness Level (TRL) also used by the European Union Horizon 2020 program [5] on a scale of 1 to 9, within which a TRL of 4 meaning the validation of functional capability in laboratory environments. Systems that are suitable for everyday use require a TRL of at least 6.

### 2.1. Methodological–Technical Aspects

Mobile assistive robots require comprehensive technical capabilities suitable for everyday use in any application [6]. Basic skills comprise autonomous, obstacle-avoiding navigation, the ability to robustly recognize, track, and identify people, and also the ability to interact with users. The concrete realization of these skills already determines the degree of robot autonomy that can be achieved in an application. Only a high degree of robot autonomy allows extensive user tests, as well as efficacy studies for which no additional technical on-site support is possible. Advanced skills are required depending on the specific training application. For example, keeping a patient constantly centered in the detection range of the gait training robot’s sensors is essential for an exact gait feature extraction and analysis [7].

Further, the analysis of the gait pattern of patients after orthopedic operations on the hip requires the extraction of gait characteristics such as equal step length, stance duration, step width, tilt of the upper body, as well as flexion and extension of the knee and hip. In ROGER, the correct use of the Forearm Crutches (FACs) in three-point gait was also analyzed, because only the correct usage of FACs relieves the operated hip and yields the desired therapeutic results. To provide the patient with immediate feedback on the gait errors made, an evaluation of the extracted features is also necessary. Furthermore, the selection of suitable robot sensors already has an influence on the methods to be used. In ROGER, due to the requirement of low hardware costs, while having the same or comparable quality as stationary laboratory systems, cost-effective sensors were preselected.

All these different basic abilities and advanced skills have to be integrated into a training application suitable for real clinical environment conditions. Therefore, the application should be specified with all user groups, which are medical professionals, therapists, and patients (see Section 4.1). The general procedure should be suitably documented if an approval as a medical device (TRL 7) is supposed to be achieved.

The assessment of the state of development from a methodological–technical point of view must be carried out by means of technical benchmarking at several development stages, for which data from test persons (TRL 3), data from patients under laboratory conditions (TRL 4), and finally, within the framework of an efficacy study on patients (TRL 5–6) are collected and evaluated (see Section 5). Such a comprehensive assessment also requires a data management plan to implement data protection and maintenance procedures, a positive vote by an ethics committee, and insurance coverage for the users.

### 2.2. User Perspective

To consider the user perspective in the development of SAR-based gait self-training, guidelines such as the MEESTAR model [8] can be used. Complementarily, fifteen ethical guidelines for the use of age appropriate assistance systems were summarized in [9], e.g., self-determination, safety, privacy, ease-of-use. Other important aspects of the guidelines include enabling social participation, equal opportunity for use, privacy, and liability. In addition, user confidence in the correctness of the method is important for the acceptance of the system. From the patient’s point of view, four aspects play an important role: usability, safety, trust, and independence.

The target group of patients addressed by the robot-assisted self-training covers a wide age range, but is dominated by seniors who have received a hip prosthesis. Therefore, the robot as a digital therapy device must be easy to operate. This benefits not only the patients, but also other users, such as therapists, who expect a device that is no more difficult to operate than a smartphone. Furthermore, patients must generally feel safe during training. Especially for initially insecure patients, the embodiment effect of a robot is an element that can create sympathy and trust.

Patients also have different daily schedules, different levels of motivation, and time windows between therapies that allow them to train on their own. A robot should make it possible to train independently and safely, even outside of therapist working hours, e.g., in the evenings or on weekends. Therefore, the training procedure and the navigation behavior of the robot must be predictable and clearly defined, the voice outputs and the menu guidance must be formulated simply and clearly in terms of content, and they must be taken into account accordingly in the design of the Graphical User Interfaces (GUIs).

From the point of view of therapists and physicians, training plans must be easy to create individually for each patient and adaptable to the changing training progress. Thus, it is also necessary that the therapists and physicians have access to the results of the actual training.

### 2.3. Health Economic Aspects

The financial benefit of robot-assisted training in inpatient care can be cost saving in the context of the case-based cost coverage (diagnosis-related group), resulting from, e.g., lower personnel expenses. Furthermore, faster throughput times of patients can also provide a financial benefit, as they lead to an increase in turnover and thus in capacity, as well. In the inpatient rehabilitation sector, the effects are more likely to be seen in the optimization of staff deployment and internal work organization. In the outpatient sector, costs are reimbursed by health insurance funds, whereby inclusion in the catalog of remedies is a prerequisite for reimbursement.

In any case, proof of the health–economic and medical–therapeutic benefits through medical efficacy studies is necessary. For the robot-assisted gait self-training presented here, a noninferiority study would already be suitable to show that the medical device is at least as good as the alternative treatment method. This can also have a supporting effect for product launch (TRL 8).

The necessary integration of the robot system into the hospital or outpatient processes requires a process analysis and evaluation, as well as intensive coordination with the relevant stakeholders. From the hospital’s point of view, the robot must be robust enough in its function that it can carry out the training autonomously without the involvement of accompanying persons, technicians, or therapists (see Section 2.1), without hindering the processes within the hospital.

## 3. State-of-the-Art

An assessment of the state-of-the-art was made on the one hand with regard to the literature and on the other hand with regard to the market, which continues to develop corresponding applications and make them available.

### 3.1. Approaches from Science

The presented SAR-assisted gait self-training with FACs mainly uses camera-based motion analysis, in which body poses are determined and evaluated by recording and evaluating 3D-based camera data. This field has developed rapidly since the release of the Microsoft Kinect as the first low-cost sensor, with tens of thousands of publications. Nevertheless, there are only a few robotics-related publications on therapy approaches that affect a person’s gait [10].

In particular, mobile robotic systems that enable gait analysis even under changing environmental conditions are of interest. In [11], a feasibility approach of using a mobile robot equipped with a depth camera to realize a skeleton tracking compared to the skeleton obtained by a stationary Vicon system (https://www.vicon.com/applications/life-sciences/gait-analysis-neuroscience-and-motor-control/, accessed on 13 September 2021) as the ground truth was presented. In [12], a gait recognition method for service robots was presented for the purpose of identifying people. Reference [13] used a robot to help in geriatric assessments, such as the “Get up & Go”-test.

In various applications, smart walkers instead of mobile robots have been used [14,15]. However, with them, the recreation of a physiological gait pattern is not possible due to the unnatural gait imposed by the walker. In addition, action recognition methods are used in clinical applications [16,17] or could be adapted for this purpose [18,19].

The special feature of the robotic system presented here is the evaluation of the gait pattern of persons using FACs. A literature search on Google Scholar provided the work of Tsuda et al. as a further project, which explicitly evaluated gait pattern performed on axillary crutches, whereby the axillary crutches were not explicitly recorded. In [20], a static camera was used to evaluate the gait pattern of a person on axillary supports, including the angle of the thighs (as an approximation of the stride length) and the acceleration of the trunk as a measure of irregularities in the gait pattern. Reference [21] brought this system to a robotic vacuum cleaner so that subjects could walk longer distances. In [22], an experimental setup in the laboratory (TRL 4) was used to determine the step lengths of the subjects walking on the axillary supports, and they were given instructions on how to adjust the step length via a display.

### 3.2. Related Products on the Market

In neurological therapy robotics, exoskeletons (orthoses) are known on the one hand and gait training robots with devices for suspending patients who have low trunk stability, such as after severe accidents or strokes, on the other. Here, the Andago system from Hocoma (CH) or the robot from Gable Systems (NL) should be mentioned (the latter is not yet available on the market). These systems do not perform camera-based evaluations to improve gait. The robot-assisted gait self-training with FACs presented in this article thus closes the gap between camera-based therapy systems and mobile gait training robots.

Further, in the area of stationary camera-based movement analysis, which is suitable for a wide range of applications and based on relatively inexpensive sensor technology, systems in the professional fitness sector such as those from Pixformance (DE) and SOLOS Mirrors (DE) should be mentioned. In addition to the camera and an evaluation unit, they have a large display for showing exercises and giving feedback to users. There are also a number of similar systems for home use such as Mirror (USA), Tonal (USA), QAIO (USA), or Vaha (DE). Motognosis GmbH (DE) offers motion-capture-based gait analysis for neurological disorders. There are also applications on smartphones such as Kaia Health (DE), an app for back training, among other things. These apps are also used in a stationary manner, i.e., the devices are fixed in position, and users perform the exercises in front of the devices in a spatially narrowly defined area.

## 4. Mobile Gait Self-Training under Real Clinical Environment Conditions

All patients of the clinical efficacy study were operated with the same surgical technique for hip replacement. Normally, with this surgical technique, patients are discharged no later than Postoperative Day 7. Depending on the course of recovery, patients may be discharged earlier. However, within the scope of the efficacy study, patients were asked to remain in the hospital until Postoperative Day 7, at which time a final gait analysis was performed. Therefore, the robot-assisted gait self-training together with the developed training application were designed for patients who were already allowed to walk unattended after hip replacement surgery from the second postoperative day with FACs in three-point gait after permission by the physicians. For each patient, the robot-assisted self-training was carried out twice a day for 5 to 10 min in addition to the clinic standard therapy until the patient was discharged between the 5th and 7th postoperative day. The duration of one session was predefined by the therapist for the patients appropriate for their state of health individually. However, patients could also end the training prematurely if they were exhausted. This resulted in a maximum number of 12 training sessions over 6 training days. Despite the seemingly low number of training sessions, the expectation of the physicians and therapists was that early walking of the patients was not only important for recovery, but also would lead to a better gait pattern by gait correction instructions, which would lead to an earlier patient discharge in turn.

Tests of the robot-assisted gait self-training took place at Waldkliniken Eisenberg, an orthopedic hospital located in Thuringia, Germany. Training sessions always took place in the same hallway in the hospital. To ensure the patient’s privacy (see requirements in Section 2.2), the starting and ending dialogues of the training (using the research platform) took place in a separate area beside the training hallway. To ensure the safety of the patient (see requirements in Section 2.2), a training hallway was chosen where clinic staff were always within hearing distance and were instructed to help the patient, if the patient were to call them.

During the first training session, a therapist instructed the patient in the use of the robot. According to the requirements in Section 2.1, the robot moved in front of the patient at a fixed distance while the patient’s gait characteristics were extracted [23] and evaluated in a rule-based manner with regard to thresholds specified by the therapist [3]. If gait errors were detected, corrective voice and GUI outputs were given to the patient. Patients were able to pause at any time during the training for which they could use chairs in the corridor. The training robot could detect such pauses and autonomously parked at a waiting position until the training resumed.

### 4.1. Training Application

In the following, the phases of a typical ROGER gait self-training session of the research platform (see Section 4.2) are outlined as described in [2]. To initiate a training, the patient has to go to the robot, log in by using a personalized RFID transponder, and take a seat in a nearby chair. To enable an interaction, the robot approaches the sitting patient and turns so that the patient can see and reach the display [24]. Then, the robot starts to interact by speech, e.g., “Hello, I am your robotic gait coach! At first, I’d like to remind you of the procedure and the focus of our training session”. In addition, the patient can optionally view a video on how to walk in three-point gait with FACs. At the end of the introduction, the patient is asked to rate her or his physical condition. After that, the robot asks, “Are you ready? After confirming on my display, I move to the hallway, and we are going start the training.” While driving to the starting position in the hallway, the training robot generates a temporarily, nonidentifying, color-based model of the patient’s clothing to recognize them among other people in the training hallway.

During the training session, the robot leads the patient a certain constant distance and keeps the patient at a suitable position in the sensoric field of view in order to continuously obtain an analyzable 3D skeleton [7]. If a deviation from the expected physiological gait pattern is detected, the training robot gives speech and GUI-based feedback to the patient, e.g., “Make sure to take equally sized steps with both legs”.

At the end of the hallway, the robot stops, turns around, and waits, while the patient walks around it and is sensed behind the robot again. After that, the robot continues guiding the patient while training. Along the hallway, chairs were placed to allow the patient to rest. If the robot detects that the patients has taken a seat, it starts approaching the patient in the same way as the robot approaches the sitting patient after initially logging in. The robot states “I notice you want to pause the training. Do you want to take a break or rather finish the training?”. If the patient decides to continue training, he/she gets up after his/her break and continues walking. In the case of finishing training, the patient will be guided to the starting position to terminate the training session with a closing dialogue, e.g., “You walked 100 m very well. For the next training, please pay attention to the usage of crutches and straighten your upper body”. After finishing the regular training time, a similar dialogue appears, as well.

The physiotherapists have access to the results of each training. According to the results, they may adjust the training time for the next training session. Depending on the way the robot is integrated into the hospital infrastructure, physiotherapists might also be able to follow the gait training session in real time.

### 4.2. Robot Platforms Used

**Figure 2:**
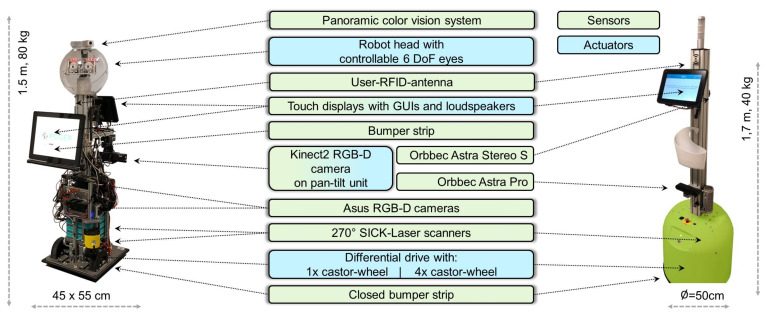
Sensors and hardware of the research platform (**left**) and product prototype platform (**right**).

To assess the robot-assisted gait self-training, two technologically different stages of training robots were used. To investigate the general technical feasibility, a research platform was used (see Figure 2, left). Based on this, the transferability of the developed methods to a prototype platform that is closer to a product and equipped with low-cost sensors and hardware was also investigated (see Figure 2, right).

The base of the research platform is a customized SCITOS G3, and the base of the product prototype platform a TORY, both developed by MetraLabs GmbH (www.metralabs.com/en, accessed on 13 September 2021). Both robots have a similar height of 1.5–1.7 m, a footprint of 0.45×0.55 m, 0.5 m as the diameter, and can reach a speed of up to 0.9 $\frac{m}{s}$.

As the primary user interface, two touch displays are mounted on the research platform at different heights, allowing standing or sitting patients to comfortably interact with the robot. To reduce costs, the product prototype platform has only one display, mounted at a height to interact when standing.

**Figure 3:**
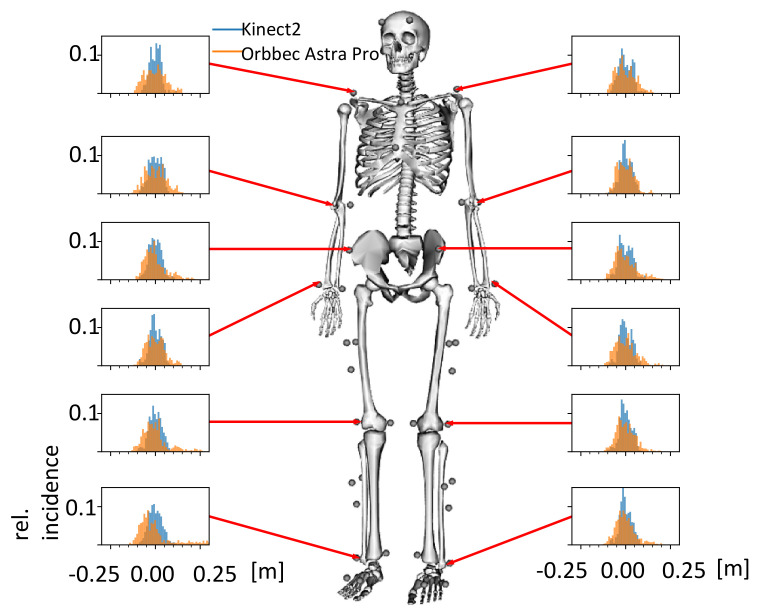
Histograms of the errors between the Kinect2 (blue) and Orbbec Astra Pro (orange) compared to a Vicon system for different joints.

The main difference in the sensors between the product prototype and the research platform is that the latter one uses a robot head with an omnicamera to detect people and distinguish the patient undergoing training from other people [25]. To assess the patient’s gait during training, a Kinect2 was mounted on a pan-tilt unit so that it can move on the research platform. Therefore, despite the Kinect2’s relatively narrow field of view of $70^{\circ }$, patients can be kept in view actively [7] even when the robot has to evade obstacles or persons that are encountered on the training track. Using only low-cost sensors, a fixed Orbbec Astra Pro camera combined with the Nuitrack SDK for skeleton estimation was utilized on the product prototype platform. The detection of the patient during the training was carried out on the product prototype platform via the skeleton captured with the Nuitrack SDK and the re-identification of the patient by an identification card that differs in color from the environment and is worn by the patient. A Kinect2, for which numerous accuracy studies are available in the literature, could not be used on the product prototype platform because Microsoft stopped its production in 2015 and a product prototype platform should contain sensors that will be available on the market in the future only. Therefore, the decision was made to use an Orbbec Astra Pro camera in combination with our preliminary accuracy studies (see Figure 3).

Accuracy studies in the gait laboratory should show whether the Orbbec Astra Pro has similar accuracy to the Kinect2, whose accuracy has already been shown to be sufficient in our preliminary studies (see also [23]) compared to the Vicon reference system (a static marker-based motion capture system). For this, Kinect2 and Orbbec Astra Pro were simultaneously attached to the robot, and the 3D skeleton points obtained by them were compared regarding the accuracy of these from the Orbbec Astra Pro to those of the Kinect2 and the Vicon system. While the standard deviations of the errors for the joint positions of the lower body estimated with the Kinect2 were between 3 and 5 cm, they were slightly higher with the Orbbec Astra Pro (see Figure 3). Despite the slightly higher error values for the Orbbec Astra Pro sensor, it was used for deployment on the product-related platform.

### 4.3. System Architectures for Both Robot Platforms

**Figure 4:**
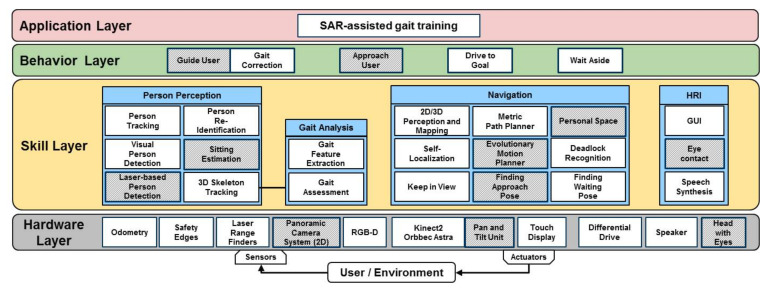
Multilayered functional system architecture of both self-training robots consisting of a Hardware Layer to control the onboard sensors and actuators, a Skill Layer with person perception, gait analysis, navigation, and HRI-specific methods and skills, a Behavior Layer comprising more complex skills, and an Application Layer implementing the whole training session. The shaded modules are used for the research platform only.

To manage the complexity of the application for the SAR-assisted gait training, a hierarchically organized software system with multiple abstraction layers was used (see Figure 4). As described for the research platform in [2,3], here, for both platforms, the robotic middleware MIRA [26] was utilized, allowing decomposing the application into modules, which can be developed and tested independently.

In the Hardware Layer, the onboard sensors and actuators used in the robot platforms (see Section 4.2) are organized.

The Skill Layer builds on the sensor information and actuator’s signals of the hardware layer to provide the core functions of the robot-assisted self-training. These core functions can be categorized into modules for navigation, person perception, gait analysis, and HRI. Only the main modules are discussed in the following. For more technical details and experimental evaluation, refer to the given references below.

Navigation: Both robot systems use similar approaches of mapping and localization, but different concepts for motion planning. The robot plans its path to the end of the hallway and adapts its speed to that of the patient so that the patient will be kept at a certain distance from the robot. The robot stops when the patient is outside its sensor detection range and continues when the patient is detected again.

Extending this, the research platform processes 3D information based on [27,28] for localization and obstacle detection. When the robot moves in a hallway of the hospital, other people (staff, patients, visitors, other bystanders) may also be present and have to be taken into account to realize a polite navigation. Therefore, these persons have to be considered in the robots motion planning methods based on a multi-objective optimization with an evolutionary algorithm [29]. The resulting motion trajectory is intended to keep the patient at a predefined distance from the robot and in the center of the Kinect2’ field of view, such that the patient is fully visible to the Kinect2 [7], but also to navigate politely by avoiding other people as much as possible.

Furthermore, the research platform uses pose finding skills to determine poses to approach and observe the patients when they take a break in a chair. These skills depend mainly on the accuracy of the input of the localization and obstacle detection module providing the current pose and the location of obstacles in the robot’s vicinity. By using a multi-objective optimization approach based on particle swarm optimization, the best poses to approach and observe the patient [24,30] can be calculated.

Person perception: Both platforms use different approaches to detect and track people, both capable of estimating the positions and velocities of persons in the robot’s vicinity. Re-identifying the patient among other persons is crucial for the training application since in the hallway, other persons may cross the robot’s path. As described in Section 4.2, the product prototype platform uses the Nuitrack SDK for person tracking and skeleton estimation and for re-identification a colored identification card.

The research platform uses a complete multimodal person tracking framework [31], which is also capable of re-identifying the patient among other detected persons. As detection modules, OpenPose [32], YOLO (https://pjreddie.com/darknet/yolo/, accessed on 13 September 2021), and a laser-based detector are able to detect persons’ legs, even when mobility aids [33], i.e., crutches, walkers, or wheelchairs, are used. Tracking the position and velocity of the detected persons is performed by utilizing a multivariate Kalman filter. The re-identification was based on a face-based approach [34] and the patient’s overall appearance by using a metric-learning approach with color and texture features [35].

Gait analysis: Both robots rely on the same methods for gait analysis, but use different sensors (see Section 4.2). Using the Kinect2 or the Orbbec Astra Pro in conjunction with the Microsoft’s SDK or the Nuitrack SDK, a fully functioning 3D skeleton tracker that robustly estimates a 25-joint skeleton (Kinect2) or 19-joint skeleton (Orbbec) in real time (both with 30 fps) was utilized.

To describe the patient’s gait, a workable approach is to analyze the time course of the 3D skeleton points. Using the extracted 3D information, gait features can be determined, e.g., step length, stance duration, step width, trunk lean, and flexion/extension of knee and hip. Furthermore, all patients walked with FACs in three-point gait, whereas the correct execution, as an important factor for the healing progress, was also analyzed using the point cloud of the depth image of the 3D camera and relating the patient’s feet to the crutch position.

Besides the extraction of gait features, these also have to be evaluated regarding a pathological gait and deviations from the expected gait. Therefore, in a previous study, the patients’ walks during training sessions were filmed by the robot and labeled by four physiotherapists regarding various deviations from physiological gait. The predefined error labels concerned step length, stance duration, step width, tilt of the upper body, flexion/extension of hip and knee joints, as well as the correct usage of the FACs. To assess related gait features, absolute values and also symmetry values, obtained by the ratio of the same feature of both legs, were used, e.g. to assess the step length, the ratio of the step length of both legs must be considered. However, the assessment of, e.g., the step width was based on an absolute value. For further details on the extracted gait features and assessment algorithms, see also [2].

HRI: Both robots have skills for displaying graphical user interfaces on the robot’s touch display(s) (as depicted in Figure 2) and a text-to-speech system (TTS, Nuance, https://www.nuance.com, accessed on 13 September 2021). On the research platform, this TTS generates spoken language in real time, allowing customizing the gait correction instructions to the patient’s needs, while the product prototype platform used the TTS’ presynthesized language.

Behaviors in the Behavior Layer realize directly observable functions of the robots by controlling the interplay of the required modules in the Skill Layer. They can be regarded as small state machines, parameterizing and coordinating the activation and deactivation of skills. Examples of behaviors are “Guide User” (using the skills, e.g., “Evolutionary Motion Planning”, “Keep in View”) and “Gait Correction” (using the skills, e.g., “Gait Feature Extraction” and “Gait Assessment”) to analyze the patients’ gait while guiding them through the hospital hallways.

The Application Layer is the top layer of the hierarchical system architecture and contains the implementation of the whole training session (see Section 4.1) as a state machine.

## 5. State of Development from a Methodological–Technical Point of View

### 5.1. Technical Benchmarking

With the two technologically different stages of the training robots, two series of user tests were carried out at the Waldkliniken Eisenberg in the period 6–8/2019 and 9/2019 (see Figure 1). In the first series of tests, two of the questions set out in Section 1 were investigated with the product prototype platform: whether the level of development achieved already enables autonomous use in everyday clinical practice (Question 1) and whether the gait pattern of patients who used additional robot-assisted gait training over several days had changed compared to patients without this additional training (Question 2). The aim of the second test series was a technical benchmarking of the methods used on the research platform, especially those that were not transferred to the prototype platform due to the stage of their development or deliberately reduced sensor technology. During both test series, a technology evaluation was carried out from the perspective of patients and therapists (see Section 6) to answer Question 3: how the use of a training robot affects the patients’ training motivation.

#### 5.1.1. Product Prototype Platform

In the period from 6–8/2019, the first and small-scale clinical efficacy study was conducted with the product prototype platform without technical on-site support. Two patient groups of a total 37 patients were formed, and their gait patterns were compared. While one group with 15 patients (20 patients planned and 5 drop-outs for standardized gait analysis in the gait laboratory on the 7th postoperative day) received the usual standard therapy until discharge from the hospital, the other group with 15 patients (17 patients planned and 2 drop-outs for standardized gait analysis in the gait laboratory on 7th postoperative day) completed an additional robot-assisted self-training for 5–10 min twice a day. The participating patients could log in to the robot at any time using a personalized RFID transponder. The first training session took place together with a therapist who instructed the patients in the use of the robot.

The data of the participating patients were entered by the therapists with their respective specific training parameters (side of surgery, duration of training) via a therapist interface. The robot itself was located on a charging station in a separate room of the clinic outside of the training times during the test period. In the morning, the room was opened by the clinic staff, and the robot was started (in the evening, the robot returned to the room to its charging station, was put into stand-by mode by the clinic staff, and the room locked). After leaving the charging station, the robot moved to its starting position in the adjacent training corridor autonomously.

As a result, the 17 patients (with drop-outs for gait analysis in the gait laboratory) completed 142 training units (8.35 training units/patient), which took place on 40 training days with a total training time of 16.1 h (7 min/training unit) and a total training distance of 34.8 km (240 m/training unit); thereby, no external intervention in the training process was necessary.

Within the framework of the small-scale clinical efficacy study, we wanted to clarify whether and which gait characteristics changed in the patients who additionally completed training with the robot. For this purpose, Waldkliniken Eisenberg carried out a standardized gait analysis in their own gait laboratory on the preoperative day and on the seventh postoperative day in conjunction with the recording of clinical scores, which are a subjective measure of perceived pain and quality of life.

The results of the efficacy study are summarized from a medical point of view in [4]. Based on literature studies, it was shown in [4] that patients after hip prosthesis surgery walk more slowly and have a lower hip range of motion than healthy individuals. In addition, ref. [4] discussed the importance of corrective gait training after hip arthroplasty surgery as an important aspect of fall prevention, as it corrects pain avoidance strategies acquired before surgery that may lead to falls.

Therefore, the results obtained in our study can be considered promising from a medical point of view. Thus, the group that received additional robotic training showed several statistically significant better postoperative gait parameters, namely knee flexion angle (important for a more physiological gait) and higher walking speed (more than 25% faster than the group without additional training), which also shortened the stance duration, including that of the operated leg.

The similar results in the clinical scores of both groups were also positive, as patients in the group with additional training did not have higher subjectively perceived pain despite the increased movement. The details of the results achieved from a medical point of view together with details to statistically significant improvement of gait parameters were shown in [4].

An evaluation of the accuracy of the recorded gait parameters and gait corrections during training was not performed due to the lack of ground truth data. However, the proof of the sufficient accuracy of the extracted gait parameters was already shown in a prestudy [2,23].

#### 5.1.2. Research Platform

During the second test series in 9/2019, investigations were carried out with the research platform on 16 training days with 22 patients. They used the robot on up to three consecutive training days for a total of 91 training units (4.14 training units/patient), with a total training time of 11.6 h (7.6 min/training unit) and a total training distance of 17.8 km (195 m/training unit).

With the technical benchmarking carried out in this context, it was possible to evaluate methods in the area of view-based recognition and autonomously finding and approaching the waiting and interaction positions, among others. For this purpose, technical support was provided on-site in order to be able to intervene in the training process in a corrective manner. In addition, the patients were asked to take several breaks in a chair in the training corridor during the training to evaluate a few of the person perception and navigation skills (sitting estimation, find approach pose, find waiting position) under field conditions.

By means of technical benchmarking, it could be shown that the patient was held at a specified distance and angle to the robot during 99.6% of the training time, even without readjustment by means of a pan-tilt unit. In 86% of the cases, it was possible to approach “sitting” patients to establish an interaction distance in such a way that an interaction with the robot display was easily possible (distance approximately 0.66 m and a 10° deviation orientation). The autonomously determined waiting positions of the robot during the patients’ breaks were chosen such that the seated patient remained in the focus of the robot sensors to detect when the patient got up again. In 5% of the cases, the selected robot position would have made access to rooms difficult or uninvolved persons would have had to avoid the robot in the corridor. These and other results were presented in detail in [3].

As in the first test series, no evaluation of the recorded gait parameters and the gait correction cues given to the patients was performed due to missing ground truth data. In future work, therapists should evaluate video excerpts from the user tests and the corresponding gait correction cues with regard to their quality, so that corrective feedback can be evaluated and improved.

## 6. State of Development from the Users’ Point of View

Both series of user tests, with the prototype product and with the research platform, were externally examined from a social science perspective by the Institute for Social Research (SIBIS GmbH, http://www.sibis-institut.de/en/, accessed on 13 September 2021). In both test series, patients rated the usability of the system on the System Usability Scale (SUS) [36], the most widely used standardized questionnaire for evaluating perceived ease-of-use. The aim was to determine whether both platforms could be used by the patients. For this purpose, the aspects of easy operation, the comprehensibility of the speech output, and the readability of the GUI displays were considered, as well as whether the robot adapted well to the walking speed of the patient and whether the robot behaved as expected. Using the SUS, the product-related platform was rated 81.2 (very good) and the research platform 90.7 (excellent). The better rating of the research platform may be due to the fact that the tests with it were conducted under supervision, the slightly different navigation behavior, or perhaps due to its different appearance in combination with the robot head and the robot eyes, which always keep the patient in view, which was positively evaluated. Further, it was shown that the patients trusted that both robots provided the correct corrective cues.

Further, the patients of both groups of the clinical efficacy study (*n* = 30) that received standardized gait analysis in the gait laboratory on the seventh postoperative day were also asked to assess their own exercise motivation outside of the training and therapy times. All patients who trained with the robot in addition to the standard therapy (*n* = 15) rated their exercise motivation as good or very good, compared to only 11 patients in the comparison group without robotic training (*n* = 15). All patients (*n* = 20) who trained with the research platform and answered the questionnaire agreed (fully) that they would exercise more often if they could use the robot for this purpose, thus confirming the positive effect of the robot on the patients’ motivation. Furthermore, in a survey of 22 patients who had also used the robot in pretests that went beyond these tests, 21 agreed that they would use the robot-assisted training independently without the presence of a therapist after being instructed.

Robot training was carried out during therapy-free intervals in the clinic routine, and 13 of 15 patients agreed (fully) that they felt very safe during robot training. Two patients answered “partly/partly”. This information confirmed the good implementation of the requirements from Section 2.2 for independent and safe training with robots. A quotation from the user survey showed that the use of the robots was very intuitive: “Anyone who can withdraw money from an ATM can also operate the ‘ROGER’ device”.

## 7. State of Development from an Economic Perspective

The technical possibilities of SAR-assisted gait self-training show that therapists can spend more time on “hands-on therapy”, e.g., muscle and joint techniques, cryotherapy, scar massages, etc., if the robot takes care of the patient’s self-training activities. This could in turn lead to a faster recovery. This hypothesis was supported by the initial results of the small-scale clinical efficacy study, which showed a significant improvement in the hip and knee joint flexibility of the patients [4].

For the prototype product platform, components were used that were as inexpensive as possible, but still suitable to be used in a medical device and therefore durable and reliable. This helps to ensure that a product can be offered in the future that is economically viable (TRL 8). With regard to integration into clinical processes, interfaces with clinic systems would be useful in order to achieve process automation.

Within the framework of the ROGER project, the first findings on medical efficacy accompanied by financial benefits for the stakeholders, the requirements for approval as a medical device, and the integration into clinical processes were collected and further explored through stakeholder interviews.

These findings on the economic perspective are all important aspects on the way from the demonstrator (TRL 6) to the finished product (TRL 8) and require further work.

## 8. Conclusions on the Questions of the Article and Outlook

Autonomous use in everyday clinical practice (Question 1): The product prototype platform ran for several weeks without technical or therapeutic support in everyday training in the hospital (see Section 5.1.1), which demonstrated sufficient robustness and autonomy of the robot used, the integrated methods especially for navigation, and of the overall application. In future work, it will be necessary to examine which of the robot skills initially implemented on the research platform can be implemented for everyday robot-assisted training without on-site support.

Improved gait pattern (Question 2): The clinical study showed no differences between the comparison groups with regard to the clinical scores recorded. However, the group with additional robotics training showed statistically significantly better gait parameters postoperatively. The clinical study so far has only covered part of the functionalities that a gait training robot has to fulfil. Thus, randomized clinical studies are still necessary to demonstrate the effectiveness of robot-assisted gait self-training on a broad scale. Further developments will also concern improved gait pattern recognition algorithms.

Training motivation (Question 3): By interviewing patients and therapists, combined with the demonstrated use of the robots over several weeks, it could be shown that the acceptance criteria of safety, usability, confidence, and independence were met. For further development with regard to the design of the platform, it is important to realize that the research platform with its distinct embodiment features was obviously perceived very positively and not at all irritating by the patients.
